# Molecular epidemiological characteristics of SARS-CoV-2 in imported cases from 2021 to 2022 in Zhejiang Province, China

**DOI:** 10.3389/fpubh.2023.1189969

**Published:** 2023-06-22

**Authors:** Biaofeng Zhou, Yi Sun, Haiyan Mao, Lingxuan Su, Yihan Lou, Hao Yan, Wenwu Yao, Honghu Chen, Yanjun Zhang

**Affiliations:** Zhejiang Provincial Center for Disease Control and Prevention, Hangzhou, Zhejiang, China

**Keywords:** SARS-CoV-2, genetic variation, imported cases, whole genome sequencing, phylogenetic analysis

## Abstract

**Backgrounds:**

The coronavirus disease 2019 (COVID-19) pandemic caused by severe acute respiratory syndrome coronavirus 2 (SARS-CoV-2) has been a global threat since 2020. The emergence of the Omicron variant in 2021, which replaced Delta as the dominant variant of concern, has had a significant adverse impact on the global economy and public health. During this period, Zhejiang Province implemented dynamic zeroing and focused on preventing imported cases. This study aimed to gain clear insight into the characteristics of imported COVID-19 cases in Zhejiang Province.

**Methods:**

We conducted a systematic molecular epidemiological analysis of 146 imported cases between July 2021 and November 2022 in Zhejiang Province. Virus samples with cycle threshold (Ct) value less than 32 were performed next generation sequencing. Basing the whole genome sequence obtained after quality control and assembly of reads, the whole genome variation map and phylogenetic tree were constructed and further analyzed.

**Results:**

Our study identified critical months and populations for surveillance, profiled the variation of various lineages, determined the evolutionary relationships among various lineages of SARS-CoV-2, and compared the results in Zhejiang with those obtained worldwide during this period.

**Conclusion:**

The continuous molecular epidemiological surveillance of imported cases of COVID-19 in Zhejiang Province during 2021 to 2022 is consistent with the global epidemic trend.

## 1. Introduction

In 2019, the severe acute respiratory syndrome coronavirus 2 (SARS-CoV-2) was first reported in Wuhan, China. SARS-CoV-2 belongs to the beta genus of coronaviruses, with a genome consisting of a single plus-stranded RNA of approximately 29.9 kb in length (1). The viral particle contains several structural proteins, among which the S protein plays a crucial role in infection by identifying and binding to the host cell receptor, angiotensin-converting enzyme 2 (ACE-2), through the receptor-binding domain (RBD) (2–4). Consequently, the S protein of SARS-CoV-2 undergoes frequent mutations leading to the emergence of novel variants with enhanced transmissibility and immune evasion capabilities.

Since 2020, SARS-CoV-2 has undergone numerous mutations, resulting in the emergence of several variants, with Delta first being reported in India (5–7) and being the predominant variant that began spreading widely in May 2021 (8). However, in November 2021, a new variant called Omicron, carrying multiple genetic mutations in the spike (S) gene making it highly transmissible and virulent, was reported in South Africa (9). The Omicron variant has rapidly spread to other countries and has triggered a global pandemic (10). In January 2022, local cases of the Omicron variant were first reported in Tianjin, China (11), which led to community transmission and subsequent outbreaks in other provinces (12). Currently, Omicron variants are a global public health concern. As of March 2023, 758,390,564 confirmed COVID-19 cases and 6,859,093 related deaths were reported worldwide.^1^

As of 24 December 2022, Mainland China had reported 397,195 confirmed cases and 5,421 deaths. From 2021 to 2022, Zhejiang Province reported minimal large-scale outbreaks, which can be attributed to its excellent outbreak prevention and control measures. The Zhejiang Provincial Center for Disease Control and Prevention (CDC) with 11 municipal-level CDCs, was capable of performing sequencing. In the event of a local outbreak from an unknown source, the municipal CDC conducts sequencing to trace its origin and sends the samples to the provincial CDC for review. The provincial CDC dispatches an expert team for immediate control and regulation of the epidemic. However, Zhejiang is vulnerable to imported infectious diseases owing to its highly developed export-oriented economy and frequent international and domestic travel. From December 2022 to April 2023, BF.7 and BA.5.2 were the predominant local cases in Zhejiang, but since April 2023, XBB variants and its sublineages have become prevalent owing to continued imported cases. The median daily incidence of the aforementioned cases in Zhejiang is 125 since April 2023. It is crucial to implement molecular epidemiological techniques to survey and analyze imported cases. This approach would allow for the early identification of potential risks, facilitate timely warning issuance, and enhance prevention and control measures to ensure public safety.

In this retrospective study, we conducted a comprehensive analysis of the data collected from 146 imported cases between July 21 and November 2022. This analysis included basic cases and whole-genome sequence information on SARS-CoV-2. Our primary objective was to investigate the molecular epidemiological characteristics of SARS-CoV-2 during this period and compare them with global monitoring results. By doing so, we aimed to identify the similarities and differences between imported cases in Zhejiang Province and those from other regions and elucidate the lineage variation of SARS-CoV-2 in imported cases with regional characteristics. Our findings provide insight into the key periods, populations, and countries that require focused monitoring of imported COVID-19 cases, which may facilitate the implementation of more targeted and personalized monitoring strategies.

## 2. Methods

### 2.1. Sample collection

In our study, patients with imported COVID-19 constituted individuals who tested positive for SARS-CoV-2 nucleic acid and had either resided in or traveled to any country or region outside mainland China within 14 days prior to testing. Our definition encompassed both symptomatic and asymptomatic individuals and the former included four types, namely mild, medium, severe, and critical (see Supplementary Table S1 for definition). During July 2021 to November 2022, nasopharyngeal swab samples from imported cases were collected by the CDCs located in various cities of Zhejiang Province, along with designated hospitals for COVID-19 diagnosis and treatment, such as Hangzhou Xixi Hospital (Supplementary Figure S2). These samples were promptly dispatched to the Zhejiang Provincial CDC for further analysis.

### 2.2. Nucleic acid extraction and qPCR detection

Viral nucleic acids were extracted from the samples using the QIAGEN RNeasy Mini Kit (Qiagen, Germany, lot 74,104). The viral load was determined through real-time fluorescent RT-PCR using the COVID-2019 nucleic acid detection kit (Shanghai Biogerm Medical Technology Co., Ltd., Shanghai, China, lot V513303) targeting the ORF1ab and N genes and the ABI 7500 machine. Samples with Ct values of <32 for both target genes were selected for further analysis.

### 2.3. Whole-genome sequencing

We selected samples for whole-genome sequencing based on the availability of specimens collected during the study period and the viral load (Ct < 32) of the RNA extracted from these specimens. Reverse transcription and multiplex PCR amplification were performed on all nucleic acid samples using the ULSEN SARS-CoV-2 whole-genome capture kit (Beijing Micro-Future Technology Co., Ltd., Beijing, China, lot V-090418-96), following the manufacturer’s instructions. The resulting DNA libraries were prepared using a Novel Coronavirus Library Construction Kit (Hangzhou Matridx Biotechnology Co., Ltd., Zhejiang, China, lot MDR004), with each sample linked to a unique barcode for sample identification. High-throughput sequencing was performed on the Illumina Nextseq 550 or MiniSeq platforms using a matched reagent kit with cycle lengths of 75, 100, or 150.

### 2.4. Genome assembly and variant calling

To ensure high-quality results, raw sequencing data were processed using the Microbiome and Virus Analysis Platform (MVP). The data were subjected to several analytical steps, including quality control, alignment, variant calling, and consensus sequence generation. First, Fastp (13) was used to trim primer sequences and filter low-quality reads. Subsequently, all clean reads were mapped to the reference genome for SARS-CoV-2 (NC_045512.2) using the Burrows-Wheeler Aligner (14). To eliminate duplicate sequences and non-uniquely mapped reads, we further filtered the data. We used bcftools (15) to obtain sequencing depth and single nucleotide variants (SNV). Finally, contigs and whole-genome sequences were assembled from the remaining mapped reads.

### 2.5. Genome consensus sequence correcting and variant classification

The consensus sequences generated by MVP analysis may contain SNV errors such as heterozygous variations, incorrect insertions, and deletions. To ensure data accuracy, we thoroughly examined the Integrative Genomics Viewer (IGV) results, which provided the distribution of each read mapped to the reference genome. In addition, we carefully analyzed the variation in each sample to comprehensively understand the potential errors in the consensus sequences. Geneious Prime software (Version 2021.2) was used to correct and edit sequences for accuracy. We only considered 146 whole-genome sequences that met the following criteria: coverage of more than 96% and completeness of the S gene without an N base. To classify the lineages of SARS-CoV-2, we used the nextclade v2.11.0 Web application (16).^2^

### 2.6. Phylogenetic analysis

Phylogenetic tree analysis constituted 147 SARS-CoV-2 nucleotide sequences. All sequences were subjected to multiple sequence alignment using MAFFT (17). Evolutionary analyses were then conducted in FastTree (18) using the maximum likelihood method, and the bootstrap consensus tree inferred from 1,000 replicates (19) was used to represent the evolutionary history of the taxa analyzed. Branches corresponding to partitions reproduced in less than 50% of the bootstrap replicates collapsed. Finally, the Newick file generated by FastTree was polished using iTOL online tools (20).^3^

## 3. Results

### 3.1. Descriptive analysis of the imported cases of COVID-19 in Zhejiang

Table 1 shows that the number of foreign male patients significantly exceeded that of female patients during this period, with a male-to-female ratio of >3:1. The age distribution of imported cases was prevalent within the age range of 20–29 years, accounting for 29.45% of all the cases. Conversely, the highest and lowest age groups had the lowest proportions, accounting for only 0.68% of all cases. Moreover, the four age groups comprising the major labor force (20–29, 30–39, 40–49, and 50–59) accounted for 92.46% of all the cases.

**Table 1:** General information on the imported cases of COVID-19 in Zhejiang Province, China.

Features	Count	Percentage
**Gender**		
Male	110	75.34%
Female	36	24.66%
**Age (years)**		
<10	1	0.68%
10–19	5	3.42%
20–29	43	29.45%
30–39	35	23.97%
40–49	26	17.81%
50–59	31	21.23%
60–69	4	2.74%
>69	1	0.68%
**Country**		
United Kingdom	13	8.90%
Japan	11	7.53%
Spain	8	5.48%
Philippines	8	5.48%
Mexico	8	5.48%
Canada	6	4.11%
Australia	5	3.42%
Indonesia	4	2.74%
Tanzania	4	2.74%
Korea	4	2.74%
Ecuador	4	2.74%
Panama	4	2.74%
France	4	2.74%
Others	63	43.15%
**Occupation**		
Education industry	32	21.92%
Business services	28	19.18%
Manufacturing industry	26	17.81%
Transport industry	20	13.70%
Social organization industry	14	9.59%
Unknown	8	5.48%
Domestic service industry	6	4.11%
Catering industry	4	2.74%
Unemployed (retirees included)	3	2.05%
Health industry	1	0.68%
Information technology industry	1	0.68%
Other	3	2.05%

The imported cases originated from 46 countries and regions worldwide, with the United Kingdom, Japan, Spain, the Philippines, and Mexico serving as the main source countries, accounting for 8.90, 7.53, 5.48, 5.48, and 5.48% of the cases, respectively. Most imported cases were in the education, business services, and manufacturing industries, accounting for 21.92%, 19.18%, and 17.81%, respectively. Students, commercial service personnel, and workers accounted for the highest proportions in each industry. Conversely, the unemployed (including retirees), medical personnel, and information service personnel had the lowest proportions, accounting for only 2.05%, 0.68%, and 0.68% of all the cases, respectively.

**Figure 1:**
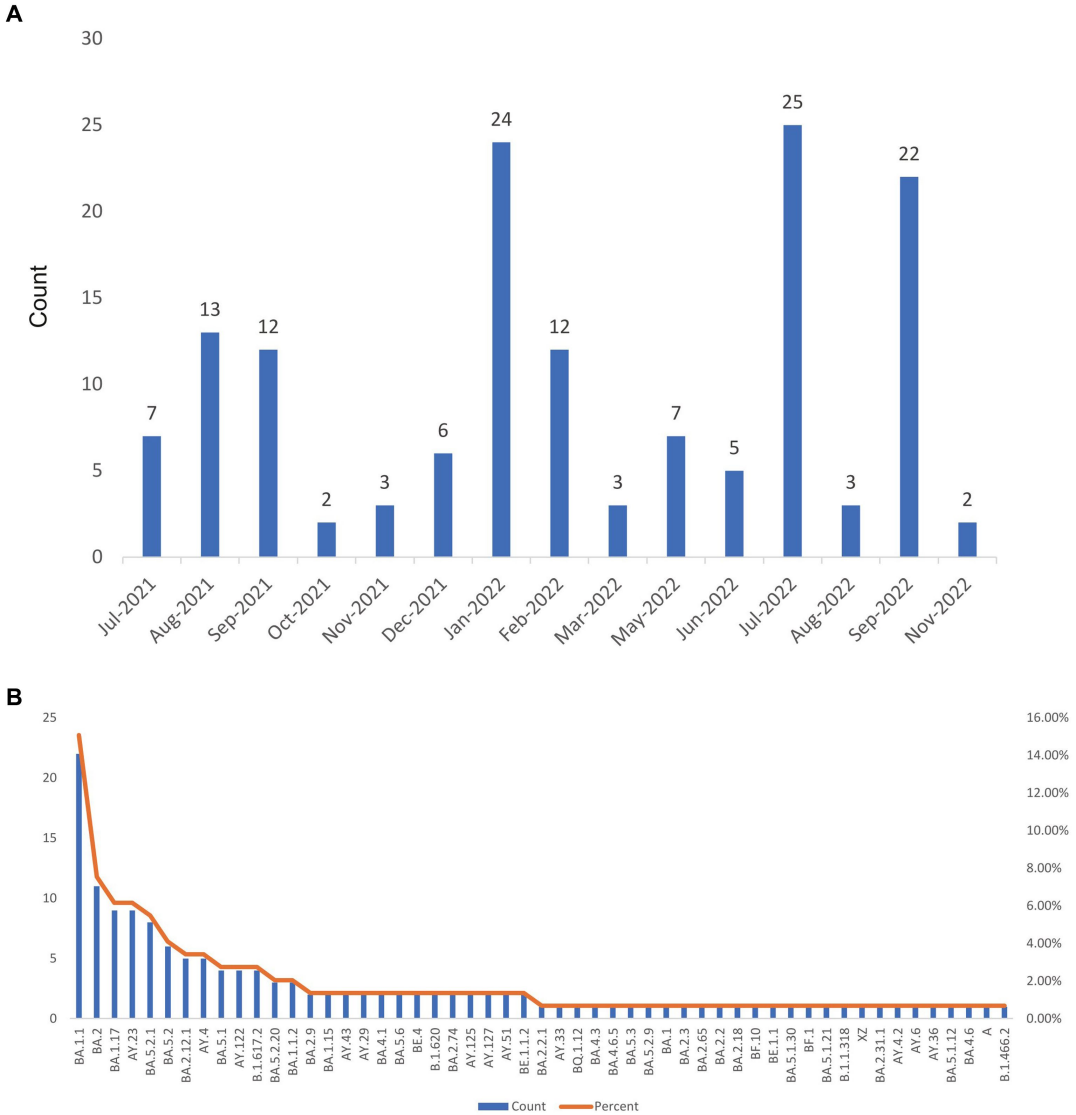
The statistic of lineages and numbers for 146 imported SARS-CoV-2 sequences. **(A)** The number of imported SARS-CoV-2 sequences per month. **(B)** The number of 53 lineages of SARS-CoV-2.

As shown in Figure 1A, there was significant variation in the number of imported cases across different months. Specifically, the number of imported cases was higher in August and September 2021, and January, July, and September 2022. Conversely, there were fewer imported cases in October and November.

### 3.2. Temporal distribution characteristics of imported SARS-CoV-2 variants

Figure 1B displays the distribution of 53 SAR-CoV-2 variants, with the BA.1.1 lineage having the highest number of sequences (15%), followed by other significant variants including BA.2 (7.53%), BA.1.17 (6.16%), AY.23 (6.16%), and BA.5.2.1 (5.48%). Among the 53 SARS-CoV-2 variants, 75% (40/53) accounted for less than 2% of all cases (less than 3). Except for a few sporadic cases, such as B.1.466.2, B.1.1.318, and B.1.620, the rest were Delta or Omicron variants. Notably, 25% (13/53) of the variants comprising a higher proportion were Delta and Omicron variants. Delta variants include AY.23, AY.4, AY.122, and B.1.617.2, while Omicron variants include BA.1.1, BA.2, BA.1.17, BA.5.2.1, BA.5.2, BA.2.12.1, BA.5.1, BA.5.2.20, and BA.1.1.2.

**Figure 2:**
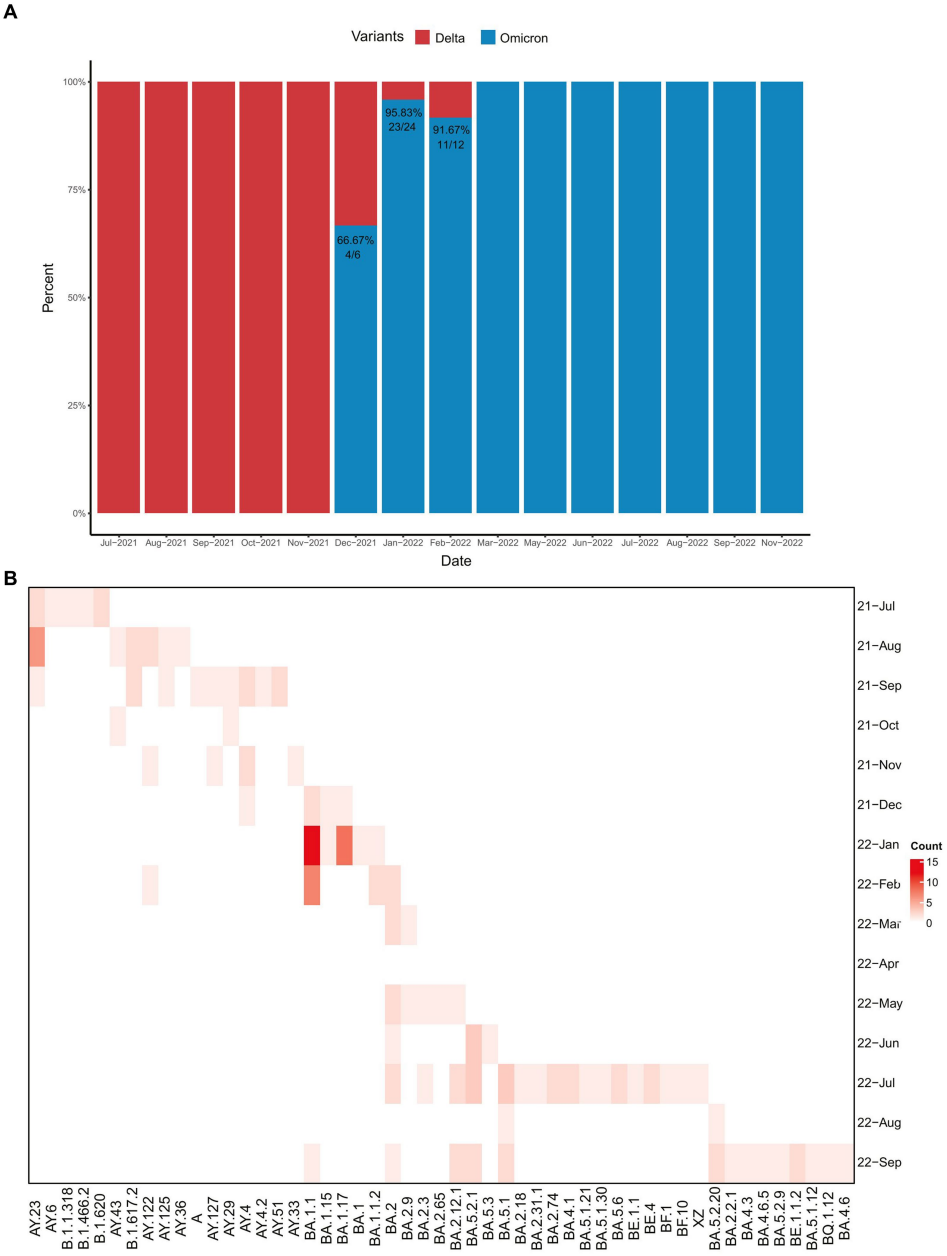
The temporal distribution patterns of 146 imported SARS-CoV-2 sequences covering 53 lineages in Zhejiang Province from July 2021 to November 2022. **(A)** Histogram of Delta and Omicron variants percentages over time. **(B)** The distribution map of SARS-CoV-2 variants detected during the monitoring period by month.

In light of these findings, we investigated the temporal distribution of Delta and Omicron variants in COVID-19 imported cases. Prior to November 2021, Delta variants were predominant in imported cases of COVID-19 in Zhejiang Province, as illustrated in Figures 2A,B. However, beginning December 2021, several Omicron variants (such as BA.1.15, BA.1.1, and BA.1.17) emerged and quickly surpassed the Delta variants, accounting for 66.7% of imported cases. This trend will persist, and is expected to reach 91% by February 2022. Subsequently, the emergence of the BA.2 variant and its subtypes was identified in February 2022 and continually detected over subsequent months, while the BA.5 variant and its sublineages occurred in June 2022 and have since been detected in ongoing surveillance efforts. In addition, numerous Omicron variants from different lineages were observed in July and November 2022. In summary, various Omicron variants gradually emerged and completely replaced the Delta variant in imported cases from February to November 2022.

### 3.3. Variation analysis of SARS-CoV-2 variants

**Figure 3:**
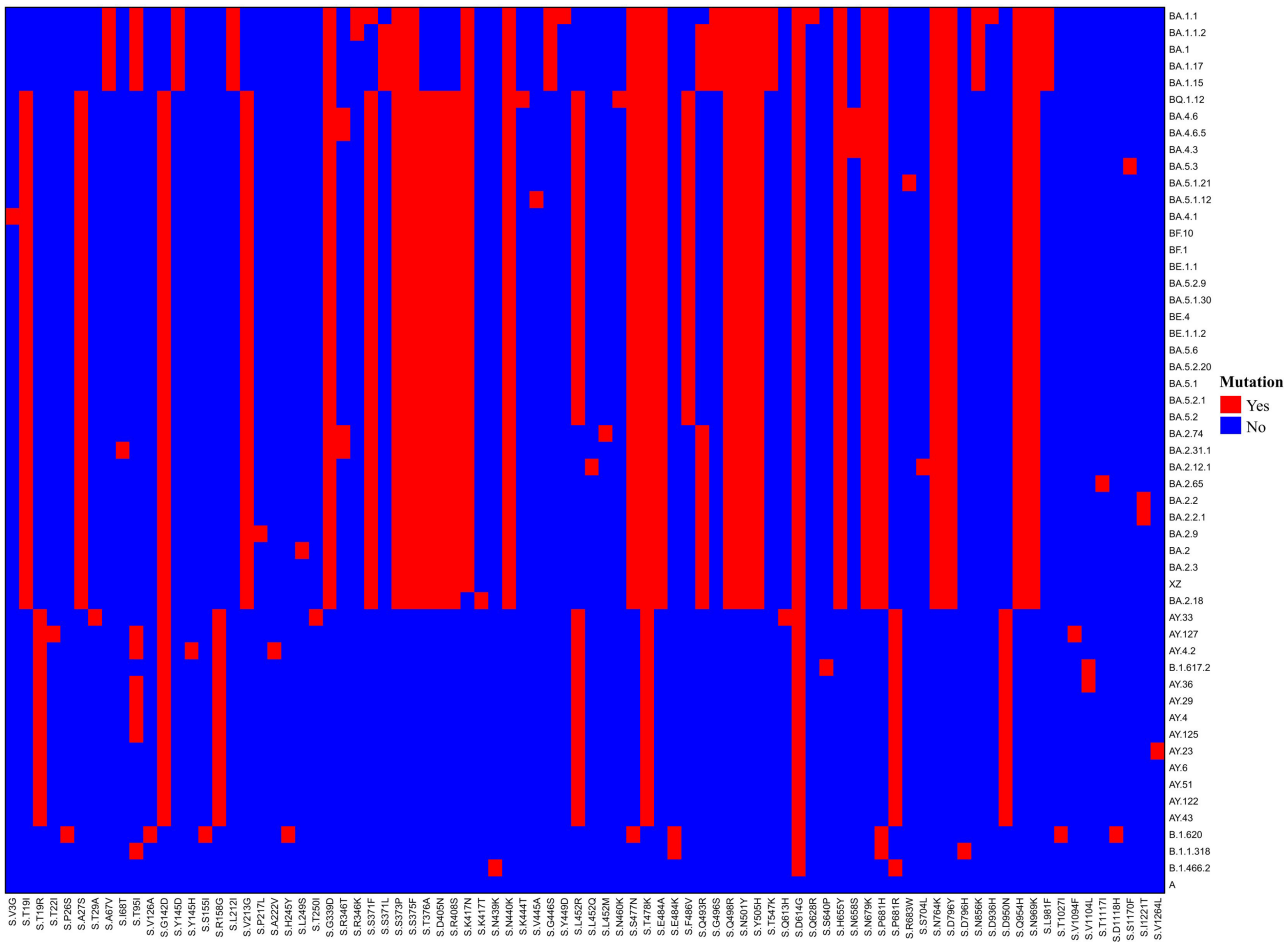
The amino acid variation map in the Spike gene covering 53 lineages from 146 imported SARS-CoV-2 sequences. For multiple sequences with identical lineages, choose the sequence with the largest number of mutations.

Results of the variation analysis of the 146 SARS-CoV-2 sequences using NC_045512.2 as a reference are presented in Figure 3. Our analysis revealed 326 amino acid mutations covering 53 variants of the SARS-CoV-2 genome (Supplementary Figure S1). We observed that ORF1a, S, and ORF1b exhibited the highest number of amino acid variations among all genes, with 89, 84, and 59 variations, accounting for 27%, 26%, and 18% of the total amino acid variation, respectively.

We focused on the S gene, which is associated with viral transmission and pathogenicity. The Delta and Omicron variants exhibited significant differences in the S gene (Figure 3), with the former containing 8–10 variations and the latter 28–33 variations. Further analysis showed that these amino acid variations were closely related to the type of variant, with some variations being widely present in all variants, such as the D614G variation, which was present in all variants except the original variant, and T478K, which was present in all Delta and Omicron variants. Additionally, certain variants, such as Delta-specific variants including D950N, P681R, T19R, and R158G, are specific to certain lineages. Specific Omicron variants outnumbered Delta variants, such as S339G, S373P, S375F, N440K, S477N, E484A, Q498R, N501Y, Y505H, H655Y, N679K, N764K, D796Y, Q954H, and N969K.

Further observation of the Omicron sublineages revealed sublineage-specific variants. For example, BA.5, BA.4, BF, BE, and their sublineages, all of which evolved from the BA.2 variant, exhibited certain stable characteristic variations, including T19I, A27S, V213G, T376A, and D405N. In contrast, BA.1 and its subtypes exhibited specific variations such as A67V, Y145D, L212I, G446S, N856K, and L981F.

Additionally, the same amino acid site in the viral genome mutates into different amino acids that are closely related to the lineages of the variants. For example, amino acid site 19 is R (Arg) in Delta variants and does not vary in BA.1 and its sublineages, but is I(Ile) in Omicron variants. The amino acid site 371 is L (Leu) in BA.1 and its sublineages (except BA.1.1) but is F (Phe) in Omicron variants. Amino acid site 484 is present as A (Ala) in Omicron variants, whereas K (Lys) is present in B.1.620 and B.1.1.318 variants. Finally, amino acid site 681 was R (Arg) in the Delta variant and H (His) in the Omicron variants.

### 3.4. Phylogenetic analyses of SARS-CoV-2

**Figure 4:**
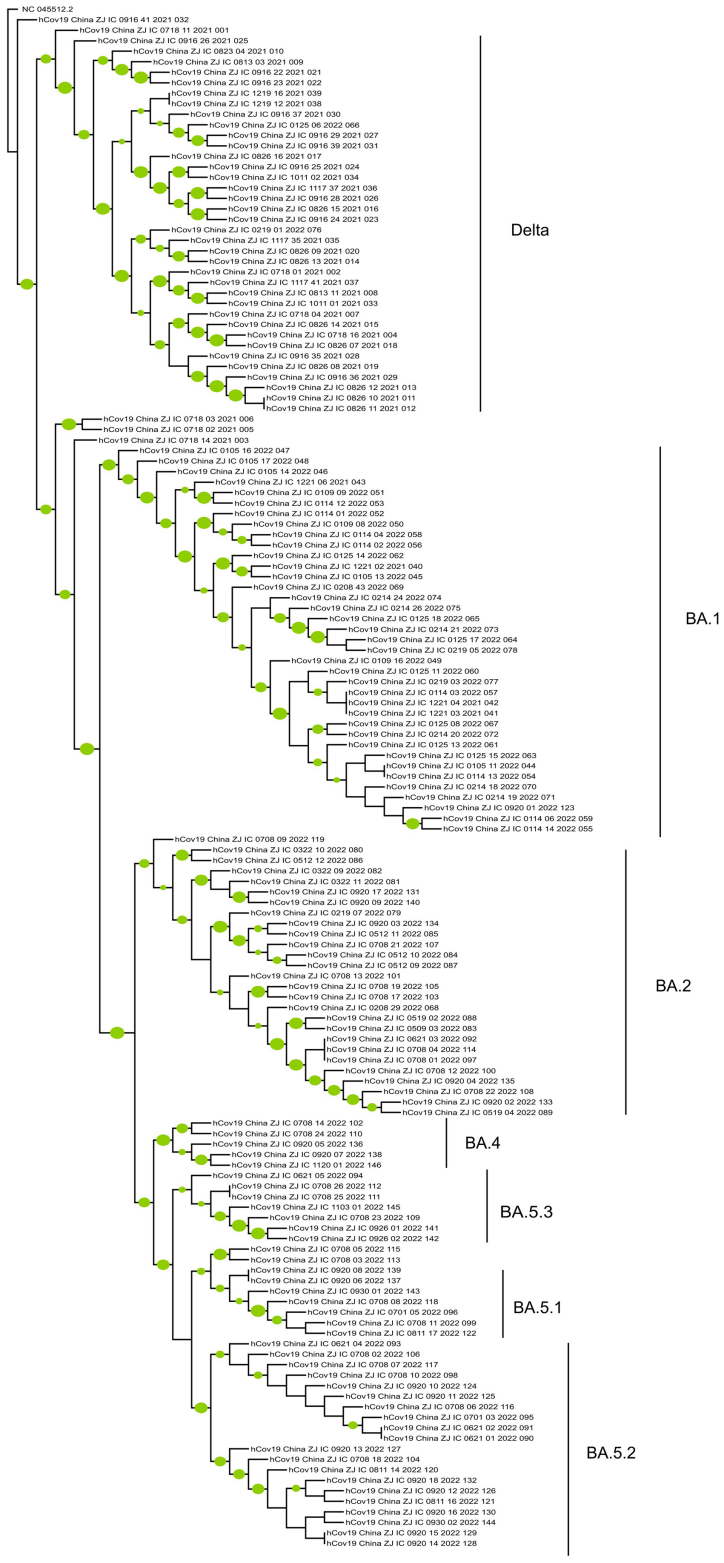
The phylogenetic tree of 147 SARS-CoV-2 genomes (include the reference sequence). The phylogenetic tree was constructed by MEGA 11 and modified by the iTOL online tool. A green circle for a branch indicates that its bootstrap value is greater than 70%, and the larger the circle, the greater its value.

The genome coverage values for the 146 whole-genome sequences in this study ranged from 98.14% to 99.84%, with an average coverage of 99.43% and a median coverage of 99.46%. Phylogenetic analysis (Figure 4) indicates that these sequences can be grouped into two main clusters: Delta and Omicron. Two subgroups were identified within the Delta cluster. One comprised BA.1.617.2 and AY.51 variants, and the other consisted of the remaining Delta variants, such as AY.23, AY.4, AY.122, and AY.125. The Omicron cluster can also be divided into two groups: BA.1 and other Omicron variants, including BA.2, BA.4, and BA.5 and their sublineages. BA.1 and its sublineages are relatively stable and show a low ability to continuously differentiate into more complex branches. The genomes of BA.2, BA.4, and BA.5, and their sublineages clustered together in the tree, showing that they were similar to each other, but significantly different from those of BA.1. Moreover, among these variants, BA.5 was further differentiated into sublineages such as BA.5.1, BA.5.2, and BA.5.3, with BA.5.2 having the largest number of cases.

## 4. Discussion

The descriptive analysis of imported COVID-19 cases in Zhejiang Province highlights a distinct population of the disease spread, with most cases being imported from the United Kingdom, Japan, and Spain in January, July, and September, with an age range of 20–29 years, likely owing to the high number of college students, business personnel, and workers traveling for business or study. The significantly higher proportion of male patients among the imported cases may be attributed to the high-risk travel behaviors associated with the COVID-19 pandemic and the high-intensity occupational characteristics of the manufacturing industry. Our findings are consistent with previous studies conducted in Beijing, where Dong et al. and Feng et al. reported a similarly relatively high proportion of males (21, 22). Moreover, Li et al. (23) observed that Spain and the United Kingdom were the primary source countries for imported cases. According to the characteristics of the COVID-19 outbreak and epidemic worldwide, there is no doubt that we should prioritize monitoring the entry of imported cases from countries or regions where the current COVID-19 outbreak or VOC appear. Based on our findings, we recommend that when surveying the inbound population for COVID-19, Zhejiang Province should pay attention to male students, workers, and business personnel traveling from the United Kingdom, Japan, Spain, and other source countries during peak importation periods in January, July, and September.

Our observations revealed that the Delta variant was dominant among imported COVID-19 cases in Zhejiang Province from July to November 2021. However, a significant shift occurred in December 2021 when the Omicron variant emerged and completely replaced Delta as the primary lineage among imported cases. These findings are consistent with those of previous reports indicating that Delta was widespread during the summer of 2021 (24–26), whereas Omicron was first identified in South Africa in November 2021 and rapidly spread worldwide (27). Additionally, our study’s epidemic times of BA.1 (December 2021), BA.2 (February 2022), and BA.5 (June 2022) were consistent with those reported by previous reports (10), considering the global spread of the virus. Overall, these results suggest that SARS-CoV-2 lineage monitoring of imported cases in Zhejiang Province is effective and provides valuable insights into the global epidemic trend of SARS-CoV-2.

Phylogenetic analyses can offer insight into the prevalence patterns and evolutionary relationships among SARS-CoV-2 variants. Between July 2021 and November 2022, the dominant SARS-CoV-2 variants shifted from Delta to Omicron, with the emergence of the Omicron subtypes BA.1 and BA.2. These subtypes subsequently evolved into the BA.2 and BA.5 sublineages. The phylogenetic tree revealed relatively numerous branches for the BA.5 sublineages, suggesting an increased competitive advantage over other subtypes. This finding is consistent with surveillance data from China since December 2022, when viral transmission rates notably accelerated owing to a change in policy. These data confirm that the two prevalent variants circulating in China, BF.7 and BA.5.2.48, both belong to the BA.5.2 subtype (28).

Our study comprehensively analyses the amino acid variations in SARS-CoV-2 variants, with a focus on the S gene, and offers insight into their molecular characteristics and evolution. The Omicron variant has a high number of mutations in the S gene, some of which have persisted throughout its evolution, suggesting its importance in viral transmission and immune escape. Notably, mutations such as S477N (29), T478K (30), N501Y (31), S371L (32), K417N (33), Q493R (34), D614G (35, 36), and others are associated with enhanced transmissibility, while mutations such as E484A (37), G446S (37), and Y505H (38) are related to improved immune evasion. Furthermore, the L452R mutation is associated with the transmission ability of SARS-CoV-2 (39) and is present in the Delta variant but absent in BA.1 and BA.2 variants. Interestingly, it reappeared in BA.5 and its sublineages. This suggests that different dominant mutations in various variants may converge to form new variants with greater transmission advantages, such as genome recombination, a concept that has been identified (38), but is yet to be researched.

The variation in sample quality from different monitoring sites was a limitation of this study, as uniform sequencing standards were not met. Additionally, the limited number of samples obtained raises concerns regarding the representativeness of the results. However, the results regarding imported cases in Zhejiang Province, including country of origin, occupation, and variant lineage, are consistent with those of previous studies, such as those by Dong et al. (21) (171 sequences), Feng et al. (22) (54 sequences in 72 cases), and Li et al. (40) (7,199 imported cases without sequences), indicating the reliability of our conclusions.

Summarily, our study demonstrates that the continuous molecular epidemiological surveillance of imported cases of COVID-19 in Zhejiang Province is consistent with the global epidemic trend. By utilizing this surveillance strategy, we can identify key populations for monitoring and detect variants of concern and their genomic variations in real time, allowing effective assessment of the epidemic risk posed by imported cases and enabling timely implementation of preventive measures.

## Data availability statement

The datasets presented in this study can be found in online repositories. The names of the repository/repositories and accession number(s) can be found in the article/Supplementary material.

## Ethics statement

The studies involving human participants were reviewed and approved by Ethical Review Committee of Zhejiang Provincial Center for Disease Control and Prevention. Written informed consent to participate in this study was provided by the participants’ legal guardian/next of kin.

## Author contributions

BZ: conceptualization, methodology, formal analysis, investigation, data curation, writing—original draft, and visualization. YS: conceptualization, investigation, resources, and writing—review and editing. HM: supervision and writing—review and editing. LS: data curation and investigation. YL, HY, WY, and HC: investigation. YZ: conceptualization and supervision. All authors contributed to the article and approved the submitted version.

## Funding

This work was supported by Key Research and Development Program of Zhejiang Province (program number 2021C03044), Major Health Science and Technology Projects of Zhejiang Province (project number WKJ-ZJ-2105), and National key R&D Program of China (project number 2021YFC2301200).

## Conflict of interest

The authors declare that the research was conducted in the absence of any commercial or financial relationships that could be construed as a potential conflict of interest.

## Publisher’s note

All claims expressed in this article are solely those of the authors and do not necessarily represent those of their affiliated organizations, or those of the publisher, the editors and the reviewers. Any product that may be evaluated in this article, or claim that may be made by its manufacturer, is not guaranteed or endorsed by the publisher.

## Footnotes

^1^ https://covid19.who.int/

^2^ https://clades.nextstrain.org/

^3^ https://itol.embl.de/

## Supplementary material

SUPPLEMENTARY TABLE 1 Clinical characteristics of 146 imported cases.

SUPPLEMENTARY TABLE 2 Genome-wide amino acid variation map of 53 lineages from 146 imported SARS-CoV-2 sequences.

SUPPLEMENTARY TABLE 3 Figure S2. Distribution map of 11 cities in Zhejiang Province.

## References

[1] Zhou P Yang X-L Wang X-G Hu B Zhang L Zhang W. A pneumonia outbreak associated with a new coronavirus of probable bat origin. Nature. (2020) 579:270–3. doi: 10.1038/s41586-020-2012-7, PMID: 32015507 PMC7095418

[2] Lan J Ge J Yu J Shan S Zhou H Fan S. Structure of the SARS-CoV-2 spike receptor-binding domain bound to the ACE2 receptor. Nature. (2020) 581:215–20. doi: 10.1038/s41586-020-2180-5, PMID: 32225176

[3] Walls AC Park Y-J Tortorici MA Wall A McGuire AT Veesler D. Structure, function, and antigenicity of the SARS-CoV-2 spike glycoprotein. Cells. (2020) 181:281–292.e6. e286. doi: 10.1016/j.cell.2020.02.058, PMID: 32155444 PMC7102599

[4] Wrapp D Wang N Corbett KS Goldsmith JA Hsieh C-L Abiona O. Cryo-EM structure of the 2019-nCoV spike in the prefusion conformation. Science. (2020) 367:1260–3. doi: 10.1126/science.abb2507, PMID: 32075877 PMC7164637

[5] Alteri C Cento V Piralla A Costabile V Tallarita M Colagrossi L. Genomic epidemiology of SARS-CoV-2 reveals multiple lineages and early spread of SARS-CoV-2 infections in Lombardy, Italy. Nat Commun. (2021) 12:434. doi: 10.1038/s41467-020-20688-x, PMID: 33469026 PMC7815831

[6] Dhar MS Marwal R Vs R Ponnusamy K Jolly B Bhoyar RC. Genomic characterization and epidemiology of an emerging SARS-CoV-2 variant in Delhi, India. Science. (2021) 374:995–9. doi: 10.1126/science.abj9932 34648303 PMC7612010

[7] Singh J Rahman SA Ehtesham NZ Hira S Hasnain SE. SARS-CoV-2 variants of concern are emerging in India. Nat Med. (2021) 27:1131–3. doi: 10.1038/s41591-021-01397-4 34045737

[8] Liu Y Rocklöv J. The reproductive number of the Delta variant of SARS-CoV-2 is far higher compared to the ancestral SARS-CoV-2 virus. J Travel Med. (2021) 28:taab124. doi: 10.1093/jtm/taab124, PMID: 34369565 PMC8436367

[9] Saxena SK Kumar S Ansari S Paweska JT Maurya VK Tripathi AK. Characterization of the novel SARS-CoV-2 Omicron (B. 1.1. 529) variant of concern and its global perspective. J Med Virol. (2022) 94:1738–44. doi: 10.1002/jmv.27524 34905235

[10] Tian D Sun Y Xu H Ye Q. The emergence and epidemic characteristics of the highly mutated SARS-CoV-2 Omicron variant. J Med Virol. (2022) 94:2376–83. doi: 10.1002/jmv.27643 35118687 PMC9015498

[11] Feng Y Shao H Gong X Song Z Xie Y Qi S. “Dynamic zero-COVID” policy and viral clearance during an Omicron wave in Tianjin, China: a city-wide retrospective observational study. BMJ Open. (2022a) 12:e066359. doi: 10.1136/bmjopen-2022-066359, PMID: 36521897 PMC9755905

[12] Yuan W Hou Y Lin Q Chen L Ren T. How China responds to Omicron. J Infect. (2022) 85:90–122. doi: 10.1016/j.jinf.2022.04.017, PMID: 35405167 PMC8993755

[13] Chen S Zhou Y Chen Y Gu J. fastp: an ultra-fast all-in-one FASTQ preprocessor. Bioinformatics. (2018) 34:i884–90. doi: 10.1093/bioinformatics/bty560, PMID: 30423086 PMC6129281

[14] Li H. (2013). Aligning sequence reads, clone sequences and assembly contigs with BWA-MEM. arXiv [preprint]. arXiv:1303.3997.

[15] Danecek P Bonfield JK Liddle J Marshall J Ohan V Pollard MO. Twelve years of SAMtools and BCFtools. Gigascience. (2021) 10:giab008. doi: 10.1093/gigascience/giab008, PMID: 33590861 PMC7931819

[16] Aksamentov I Roemer C Hodcroft EB Neher RA. Nextclade: clade assignment, mutation calling and quality control for viral genomes. J Open Source Softw. (2021) 6:3773. doi: 10.21105/joss.03773

[17] Katoh K Standley DM. MAFFT multiple sequence alignment software version 7: improvements in performance and usability. Mol Biol Evol. (2013) 30:772–80. doi: 10.1093/molbev/mst010, PMID: 23329690 PMC3603318

[18] Price MN Dehal PS Arkin AP. FastTree 2–approximately maximum-likelihood trees for large alignments. PLoS One. (2010) 5:e9490. doi: 10.1371/journal.pone.0009490, PMID: 20224823 PMC2835736

[19] Felsenstein J. Confidence limits on phylogenies: an approach using the bootstrap. Evolution. (1985) 39:783–91. doi: 10.2307/2408678, PMID: 28561359

[20] Letunic I Bork P. Interactive tree of life (iTOL) v5: an online tool for phylogenetic tree display and annotation. Nucleic Acids Res. (2021) 49:W293–6. doi: 10.1093/nar/gkab301, PMID: 33885785 PMC8265157

[21] Dong S Wang X Zhao H Wang Y Liu B Liu Y. Epidemiological characteristics of imported COVID-19 cases in Beijing. Zhonghua Liu Xing Bing Xue Za Zhi= Zhonghua Liuxingbingxue Zazhi. (2022) 43:478–82. doi: 10.3760/cma.j.cn112338-20211213-00975, PMID: 35443300

[22] Feng Z Cui S Lyu B Liang Z Li F Shen L. Genomic characteristics of SARS-CoV-2 in Beijing, China, 2021. Biosaf Health. (2022b) 4:253–7. doi: 10.1016/j.bsheal.2022.04.006, PMID: 35578696 PMC9095075

[23] Li L Ma C-J Chang Y-F Yang S-Y Tang Y-X Wang L-H. The characteristics of overseas imported COVID-19 cases and the effectiveness of screening strategies in Beijing, China. BMC Infect Dis. (2022a) 22:1–8. doi: 10.1186/s12879-021-06998-5 35039000 PMC8762985

[24] Giovanetti M Fonseca V Wilkinson E Tegally H San EJ Althaus CL. Replacement of the gamma by the Delta variant in Brazil: impact of lineage displacement on the ongoing pandemic. Virus Evol. (2022) 8:veac024. doi: 10.1093/ve/veac024, PMID: 35371559 PMC8971541

[25] Nanduri S Pilishvili T Derado G Soe MM Dollard P Wu H. Effectiveness of Pfizer-BioNTech and Moderna vaccines in preventing SARS-CoV-2 infection among nursing home residents before and during widespread circulation of the SARS-CoV-2 B. 1.617. 2 (Delta) variant—National Healthcare Safety Network, March 1–August 1, 2021. Morb Mortal Wkly Rep. (2021) 70:1163. doi: 10.15585/mmwr.mm7034e3 PMC8389386 34437519

[26] Tegally H. Wilkinson E. Althaus C.L. Giovanetti M. San J.E. Giandhari J.. (2021). Rapid replacement of the beta variant by the Delta variant in South Africa. MedRxiv [Preprint], MedRxiv 2021.2009. 2023.21264018.

[27] Viana R Moyo S Amoako DG Tegally H Scheepers C Althaus CL. Rapid epidemic expansion of the SARS-CoV-2 Omicron variant in Southern Africa. Nature. (2022) 603:679–86. doi: 10.1038/s41586-022-04411-y, PMID: 35042229 PMC8942855

[28] Pan Y Wang L Feng Z Xu H Li F Shen Y. Characterisation of SARS-CoV-2 variants in Beijing during 2022: an epidemiological and phylogenetic analysis. Lancet. (2023) 401:664–72. doi: 10.1016/S0140-6736(23)00129-0, PMID: 36773619 PMC9949854

[29] Liu Z VanBlargan LA Bloyet L-M Rothlauf PW Chen RE Stumpf S. Identification of SARS-CoV-2 spike mutations that attenuate monoclonal and serum antibody neutralization. Cell Host Microbe. (2021) 29:477–488. e474. doi: 10.1016/j.chom.2021.01.014, PMID: 33535027 PMC7839837

[30] Cherian S Potdar V Jadhav S Yadav P Gupta N Das M. SARS-CoV-2 spike mutations, L452R, T478K, E484Q, and P681R, in the second wave of COVID-19 in Maharashtra, India. Microorganisms. (2021) 9:1542. doi: 10.3390/microorganisms9071542, PMID: 34361977 PMC8307577

[31] Ali F Kasry A Amin M. The new SARS-CoV-2 strain shows a stronger binding affinity to ACE2 due to N501Y mutant. Med Drug Discov. (2021) 10:100086. doi: 10.1016/j.medidd.2021.100086, PMID: 33681755 PMC7923861

[32] Lan J He X Ren Y Wang Z Zhou H Fan S. Structural insights into the SARS-CoV-2 Omicron RBD-ACE2 interaction. Cell Res. (2022) 32:593–5. doi: 10.1038/s41422-022-00644-8, PMID: 35418218 PMC9007263

[33] Khan A Zia T Suleman M Khan T Ali SS Abbasi AA. Higher infectivity of the SARS-CoV-2 new variants is associated with K417N/T, E484K, and N501Y mutants: an insight from structural data. J Cell Physiol. (2021) 236:7045–57. doi: 10.1002/jcp.30367, PMID: 33755190 PMC8251074

[34] Focosi D Novazzi F Genoni A Dentali F Dalla Gasperina D Baj A. Emergence of SARS-COV-2 spike protein escape mutation Q493R after treatment for COVID-19. Emerg Infect Dis. (2021) 27:2728–31. doi: 10.3201/eid2710.211538, PMID: 34314668 PMC8462334

[35] Volz E Hill V McCrone JT Price A Jorgensen D O’Toole Á. Evaluating the effects of SARS-CoV-2 spike mutation D614G on transmissibility and pathogenicity. Cells. (2021) 184:64–75.e11. e11. doi: 10.1016/j.cell.2020.11.020, PMID: 33275900 PMC7674007

[36] Zhou B Thao TTN Hoffmann D Taddeo A Ebert N Labroussaa F. SARS-CoV-2 spike D614G change enhances replication and transmission. Nature. (2021) 592:122–7. doi: 10.1038/s41586-021-03361-1, PMID: 33636719

[37] Cui Z Liu P Wang N Wang L Fan K Zhu Q. Structural and functional characterizations of infectivity and immune evasion of SARS-CoV-2 Omicron. Cells. (2022) 185:860–871.e13. e813. doi: 10.1016/j.cell.2022.01.019 PMC8786603 35120603

[38] Ou J Lan W Wu X Zhao T Duan B Yang P. Tracking SARS-CoV-2 Omicron diverse spike gene mutations identifies multiple inter-variant recombination events. Signal Transduct Target Ther. (2022) 7:138. doi: 10.1038/s41392-022-00992-2, PMID: 35474215 PMC9039610

[39] Deng X Garcia-Knight MA Khalid MM Servellita V Wang C Morris MK. Transmission, infectivity, and neutralization of a spike L452R SARS-CoV-2 variant. Cells. (2021) 184:3426–3437.e8. e3428. doi: 10.1016/j.cell.2021.04.025, PMID: 33991487 PMC8057738

[40] Li Z Li Y Chen Q Jiang X Yang X Qin Y. Time distribution of positive nucleic acid detection in imported cases infected with SARS-CoV-2 in China. Zhonghua Liu Xing Bing Xue Za Zhi= Zhonghua Liuxingbingxue Zazhi. (2022b) 43:183–8. doi: 10.3760/cma.j.cn112338-20211108-00858, PMID: 35184482

