# Supplementary figures and images for: Molecular epidemiological characteristics of SARS-CoV-2 in imported cases from 2021 to 2022 in Zhejiang Province, China
Source: Front Public Health. 2023 Jun 22;11:1189969. doi: 10.3389/fpubh.2023.1189969 (PMC10323361; doi:10.3389/fpubh.2023.1189969)

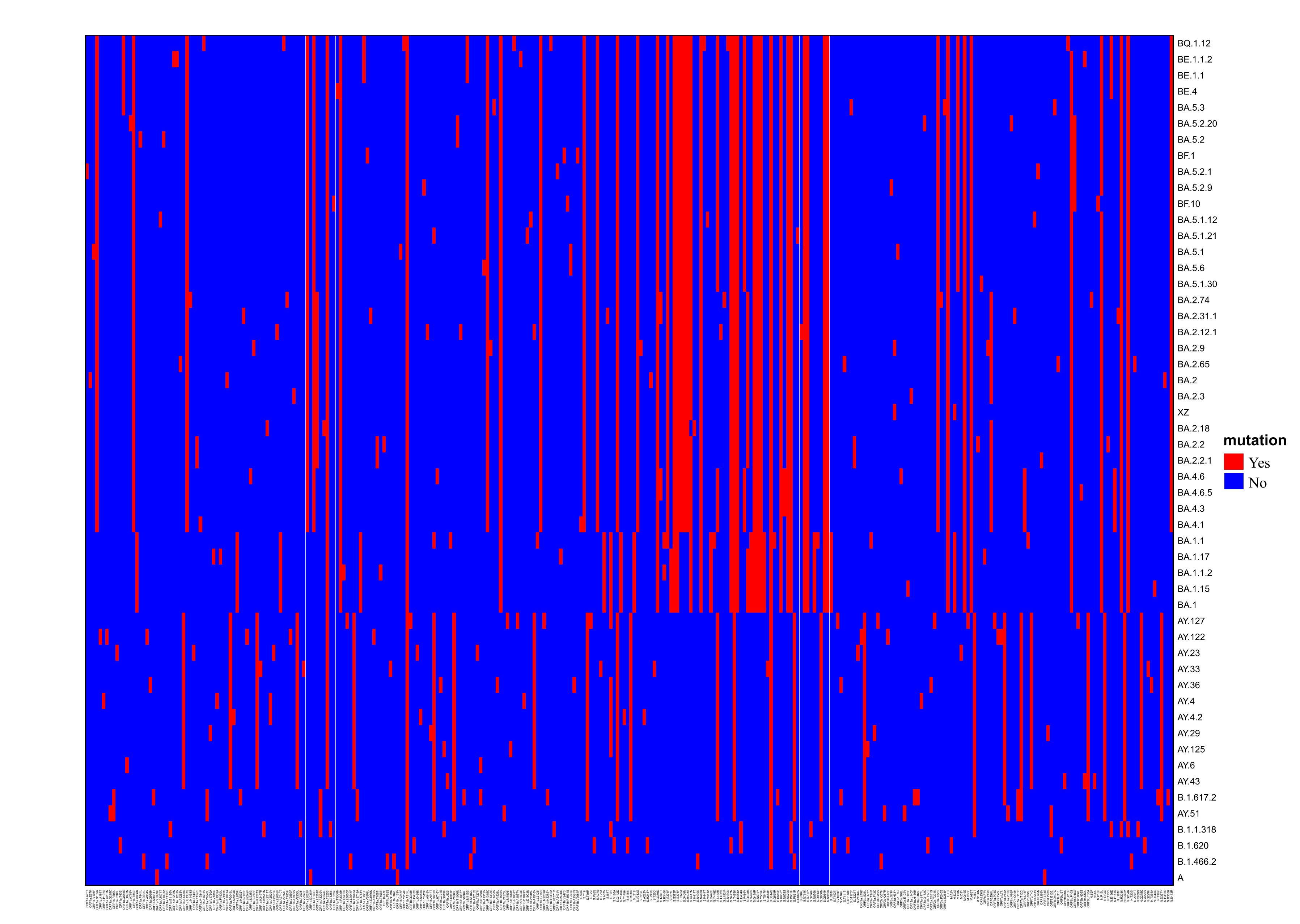

Supplement: SUPPLEMENTARY TABLE 2 — Genome-wide amino acid variation map of 53 lineages from 146 imported SARS-CoV-2 sequences. [file Image_1.JPEG]

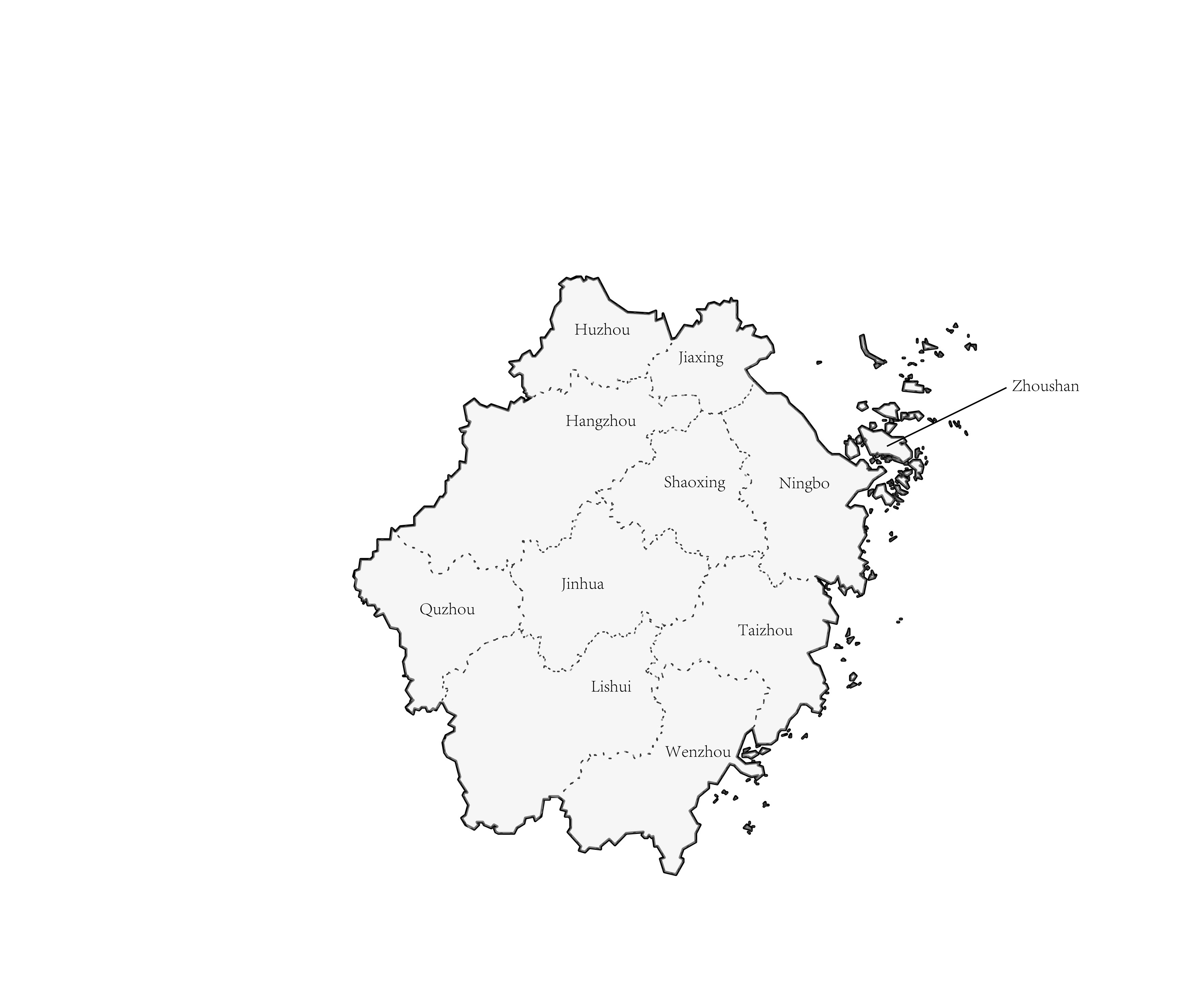

Supplement: SUPPLEMENTARY TABLE 3 — Figure S2. Distribution map of 11 cities in Zhejiang Province. [file Image_2.JPEG]
